# Effect of Thermal Aging on the Interfacial Reaction Behavior and Failure Mechanism of Ni-xCu/Sn Soldering Joints under Shear Loading

**DOI:** 10.3390/ma16155253

**Published:** 2023-07-26

**Authors:** Zhigang Li, Kai Cheng, Jiajun Liu, Yigang He, Yong Xiao

**Affiliations:** 1 School of Electronic Engineering and Automation, Hefei University of Technology, Hefei 230009, China; leiyu27@126.com (Z.L.);; 2 School of Materials Science and Engineering, Wuhan University of Technology, Wuhan 430070, China; ck_9804@163.com (K.C.);

**Keywords:** Ni-xCu/Sn soldering joints, solid-state reaction, intermetallic compounds, thermal aging, mechanical properties

## Abstract

Ni-xCu/Sn soldering joints were aged at 200 °C, and the microstructure evolution and mechanical properties during the solid-state reaction were studied under shear loading. Results showed that the intermetallic compounds (IMCs) exhibited a Cu content-dependent transformation from the (Ni,Cu)_3_Sn_4_ phase to the (Cu,Ni)_6_Sn_5_ phase at the Ni-xCu/Sn interface. Furthermore, a Cu_3_Sn layer was observed exclusively at the Cu/Sn interface. The shear strength of the soldering joints after thermal aging exhibited an initial decrease followed by an increase, except for a significant enhancement at the Cu content of 60 wt.%. In addition, the evolution law of mechanical properties and failure mechanism of the thermal aging joints were elucidated based on the fracture microstructure and the fracture curve of the joints.

## 1. Introduction

Propelled by the market demand for electric vehicles and portable electronics, the application of high-power and integrated chips led to an elevation in the service temperature of micro-solder joints [1,2,3]. Cu/Sn micro-solder joints were widely used as the interconnection system in 3D packaging, and the intermetallic compounds (IMCs) generated at the Cu/Sn interface usually consisted of a Cu_6_Sn_5_ layer and a Cu_3_Sn layer with Kirkendall voids [4,5,6]. However, the higher service temperature exacerbated the phase transition from Cu_6_Sn_5_ to Cu_3_Sn within the micro-solder joints, resulting in the development of a porous Cu_3_Sn layer [7,8,9,10]. These defects significantly impaired the electrical interconnection, signal transmission, and mechanical properties of the micro-solder joints.

To impede the growth of the Cu_3_Sn layer, current methods involved incorporating a minor Ni element into the Sn-based solder [11,12,13] or electroplating a Ni barrier layer on the surface of the Cu substrate [14,15,16,17]. These approaches effectively delayed the growth rate of the Cu_3_Sn layer. However, once the Ni element or Ni plating layer was depleted, the Cu_3_Sn layer still developed at the interface. In comparison, incorporating Ni into the Cu substrate allowed for stable growth of the reaction phase at the Cu/Sn interface. For instance, Baheti et al. [18] found that a Cu-xNi/Sn interface with a Ni content exceeding 3 at.% solely yielded a single phase of (Cu,Ni)_6_Sn_5_ without the presence of the Cu_3_Sn phase. It is noteworthy that previous studies on the Cu-Ni/Sn reactive systems predominantly concentrated on Ni content below 25 wt.%, with emphasis on the morphological changes in the single phase (Cu,Ni)_6_Sn_5_ [19,20,21]. Systematic studies to explore the composition and evolution behavior of the IMCs at the Cu-Ni/Sn interface, particularly for Cu substrates with varying Ni content, were insufficient. Furthermore, the impact of Cu incorporation on the microstructure and mechanical characteristics of Ni-Cu/Sn reactive systems when Ni was employed as the fundamental metal has been scarcely reported on.

In this study, Ni-xCu/Sn soldering joints were aged at 200 °C, and the microstructure evolution and mechanical properties during the solid-state reaction were studied with varying Cu content and aging time. Additionally, the progression of mechanical properties and failure mechanisms of Ni-xCu/Sn/Ni-xCu soldering joints were analyzed under thermal load. This study provides potential guidance for the composition optimization of micro-soldering substrates in high-power chips.

## 2. Materials and Methods

Ni-xCu alloys were synthesized via induction melting, incorporating varying Cu contents ranging from 0 to 100 wt.% in increments of 10 wt.%. Cu and Ni blocks, with a purity of 99.9%, were selected as the raw materials and individually placed into graphite crucibles according to the predetermined mass percentages. The blocks were melted under an argon-protective atmosphere. After additional homogenization treatment at 800 °C for 8 h, the deviation between the detected and intended Cu values was within 1 wt.%. The soldering surfaces were polished to a uniform 5000 mesh, and the Ni-xCu alloys were divided into 0.5 mm thick sheets and Φ5 mm × 3 mm cylinders using wire-cutting techniques.

**Figure 1:**
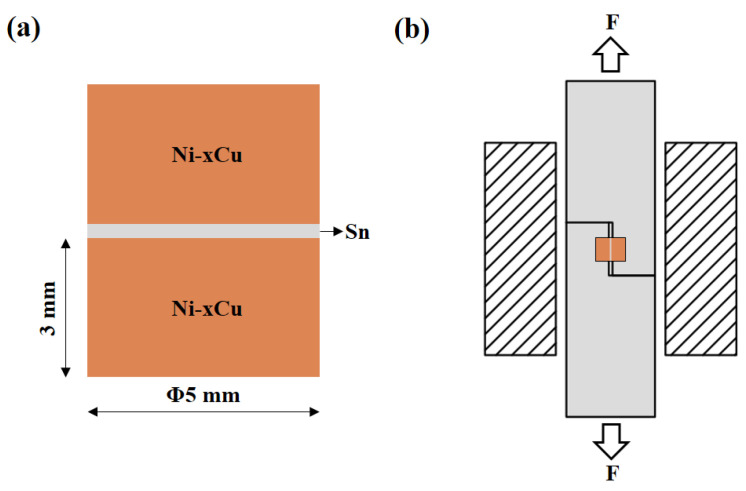
The assembly diagrams for (**a**) the shear joint and (**b**) the mechanical properties test.

A high-purity Sn ingot (99.99%) was rolled into a 30 μm thick solder strip and cut into circular discs with a 5 mm diameter. These solder discs were positioned onto the surfaces of Ni-xCu alloy sheets or assembled into a Ni-xCu/Sn/Ni-xCu sandwich structure (as shown in Figure 1a). The specimens were soldered in a vacuum furnace at 260 °C for 30 min, followed by subsequent aging at 200 °C for 24, 48 and 72 h, respectively. After the aging process, the specimens were cooled to room temperature.

The Ni-xCu/Sn aging joints and shear fracture joints were embedded in cold inlay resin, followed by grounding and polishing to observe the microstructure of the IMCs. The electron probe micro analyzer (EPMA, JEOL JXA-8230), coupled with an energy dispersive X-ray spectrometer (EDS, INCAX-ACT), was employed to characterize the morphology and component of the IMCs. The electron backscatter diffraction machine (SEM/EBSD, JEOL JSM-7001 F) was employed to analyze the phase structure, distribution, and grain size of the IMCs. Additionally, the shear testing machine (MTS-E44.104) was employed to obtain the mechanical properties of the joints, and it was conducted at ambient temperature with a shear rate of 1 mm per minute. Figure 1b shows the assembly diagram for testing the mechanical properties of the joints. To ensure reliable results, specimens for each parameter need to be tested 5 times to obtain an average value.

## 3. Results and Discussion

### 3.1. Microstructure and Phase Components

**Figure 2:**
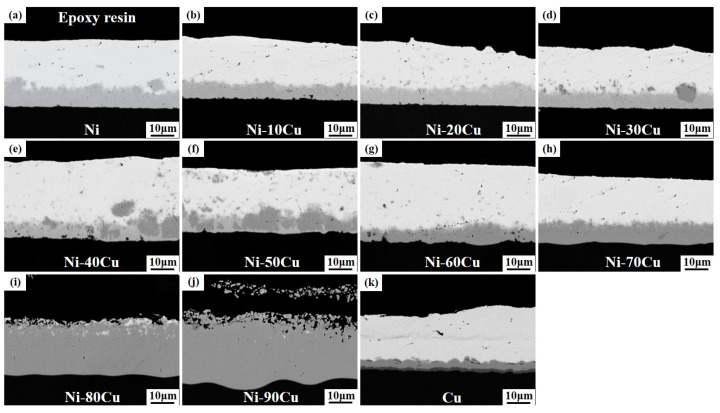
The microstructure images of the Ni-xCu/Sn joints aged for 24 h: (**a**) 0, (**b**) 10, (**c**) 20, (**d**) 30, (**e**) 40, (**f**) 50, (**g**) 60, (**h**) 70, (**i**) 80, (**j**) 90, (**k**) 100.

Figure 2 shows the microstructure images of the Ni-xCu/Sn joints aged for 24 h. The microstructure and thickness of the IMC layer exhibited a strong correlation with the Cu content present in the Ni-xCu alloys. The IMCs exhibit a continuous layer at the Ni-xCu/Sn interface as the Cu content ≤ 20 wt.% (Figure 2a–c), while particles with deeper contrast start to emerge on this continuous layer as the Cu content increases to 30–50 wt.% (Figure 2d–f). The particle count grows with higher Cu content, gradually replacing the continuous IMC layer and tending to form a continuous pattern. A reversion to a continuous layer of interfacial IMCs is observed at a Cu content of 60 and 70 wt.% (Figure 2g,h). With a further increase in Cu content to 80 and 90 wt.% (Figure 2i,j), the Sn solder is nearly depleted, resulting in the appearance of holes on the surface of the IMC layer. Additionally, when a pure Cu substrate is used (Figure 2k), the continuous IMC layer exhibits a scalloped morphology, accompanied by the presence of two contrasting regions at the Cu/Sn interface. Furthermore, except for a significant increase in the IMC layer thickness at Cu contents of 80 and 90 wt.%, there are no significant differences in the other samples.

**Figure 3:**
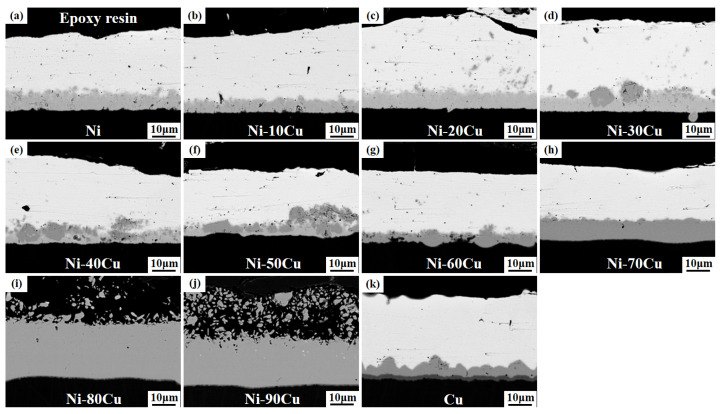
The microstructure images of the Ni-xCu/Sn joints aged for 48 h: (**a**) 0, (**b**) 10, (**c**) 20, (**d**) 30, (**e**) 40, (**f**) 50, (**g**) 60, (**h**) 70, (**i**) 80, (**j**) 90, (**k**) 100.

**Figure 4:**
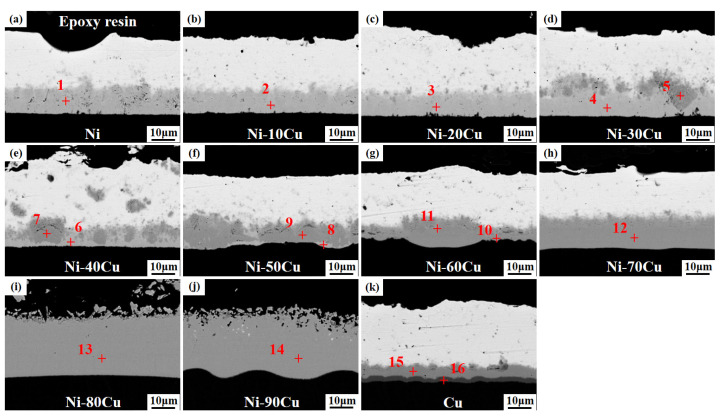
The microstructure images of the Ni-xCu/Sn joints aged for 72 h: (**a**) 0, (**b**) 10, (**c**) 20, (**d**) 30, (**e**) 40, (**f**) 50, (**g**) 60, (**h**) 70, (**i**) 80, (**j**) 90, (**k**) 100.

Figure 3 shows the microstructure images of the Ni-xCu/Sn joints aged for 48 h. The microstructure and IMC layer thickness of each sample show negligible changes with the extension of the aging time. Figure 4 shows the microstructure images of the Ni-xCu/Sn joints aged for 72 h. With the aging time further extended, the microstructure and IMC layer thickness of each sample continue to show minimal alterations, albeit with a slight increment in thickness. The above phenomenon indicates that the microstructure of the IMC layer tended to stabilize.

Table 1 exhibits the EDS detection results on the designated positions in Figure 4. In the case of pure Ni metal/Sn joints, the IMC layer can be referred to as the characteristic Ni_3_Sn_4_ phase (position 1) [22]. When the Cu content of Ni metal ≤20 wt.%, the IMCs maintain the Ni_3_Sn_4_ phase structure, but there is a minor substitution of Ni with Cu, resulting in the formation of (Ni,Cu)_3_Sn_4_ phase (positions 2 and 3) [23]. With an increase of Cu content to 30–50 wt.%, the particles formed on the (Ni,Cu)_3_Sn_4_ phase exhibit a structural transition to the Cu_6_Sn_5_ phase, wherein Ni atoms partially replace Cu atoms and manifests as the (Cu,Ni)_6_Sn_5_ phase (positions 4–9) [24]. Once the Cu content reaches ≥60 wt.%, it can be deduced that the IMCs primarily comprise the (Cu,Ni)_6_Sn_5_ phase (positions 10–14). In addition, in the case of pure Cu metal/Sn joints, interfacial IMCs are composed of the conventional Cu_6_Sn_5_ phase and Cu_3_Sn phase (positions 15 and 16) [25].

**Figure 5:**
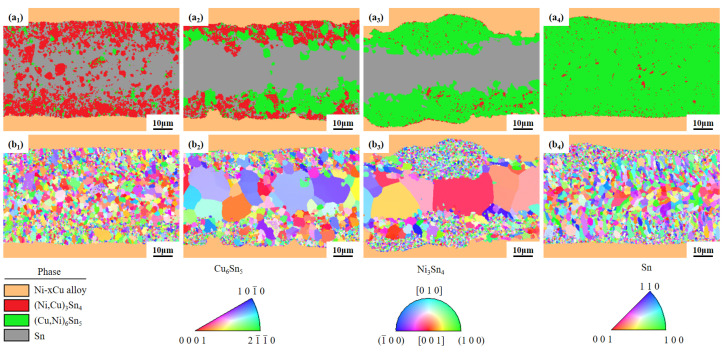
EBSD patterns for the distribution of (**a**) phases and (**b**) grains in the Ni-xCu/Sn/Ni-xCu joints aged for 48 h: (1) 20, (2) 40, (3) 60, (4) 80.

The phase structure and distribution features of the Ni-xCu/Sn/Ni-xCu joints under thermal aging were further studied based on the EBSD characterization method. Figure 5 exhibits the EBSD patterns for the distribution of phases and grains in the Ni-xCu/Sn/Ni-xCu (x = 20, 40, 60, and 80 wt.%) joints aged for 48 h. Based on the phase distribution patterns (Figure 5(a_1_–a_4_)), the joint with a Cu content of 20 wt.% predominantly exhibits the (Ni,Cu)_3_Sn_4_ phase, accompanied by a minor presence of the (Cu,Ni)_6_Sn_5_ phase, along with noticeable exfoliation within the Sn solder. As the Cu content increases to 40 wt.%, there is a corresponding rise in the proportion of the (Cu,Ni)_6_Sn_5_ phase, primarily observed as large particles located above the (Ni,Cu)_3_Sn_4_ layer. In addition, the exfoliation of reactants into the Sn solder is significantly reduced. At Cu content levels of 60 and 80 wt.%, the joints primarily consist of the (Cu,Ni)_6_Sn_5_ phase, with a minor presence of the (Ni,Cu)_3_Sn_4_ phase, and a full-IMC joint is formed at 80 wt.% Cu content.

According to the grain distribution patterns (Figure 5(b_1_–b_4_)), the joint with a Cu content of 20 wt.% exhibits fine grain size for the (Ni,Cu)_3_Sn_4_ phase and Sn solder, with a smaller grain size observed at the interface compared to the center of the weld. The grain size for the (Ni,Cu)_3_Sn_4_ phase remains fine, and the (Cu,Ni)_6_Sn_5_ phase corresponds to its particle size at a content of 40 wt.%, while the Sn solder presents as coarse grains. The Sn solder in the joint with a Cu content of 60 wt.% remains coarse grains, and the grain size of the (Cu,Ni)_6_Sn_5_ phase is significantly refined near the interface but larger near the Sn solder. The grain size is relatively small in the full-IMC joint with a Cu content of 80 wt.%, but it is also smaller at the interface compared to the center of the weld.

The aforementioned findings suggest a transformation of the reactants in the Ni-xCu/Sn/Ni-xCu soldering joints from the (Ni,Cu)_3_Sn_4_ phase to the (Cu,Ni)_6_Sn_5_ phase as the joints undergo thermal aging, with the Cu content playing a crucial role in this evolution. Additionally, the distribution behavior of the reactants has an impact on the grain size of the Sn solder. Therefore, the phase structure and distribution features of the reactants on the mechanical properties of the joints need further study.

### 3.2. Mechanical Properties and Failure Mechanism of the Joints

**Figure 6:**
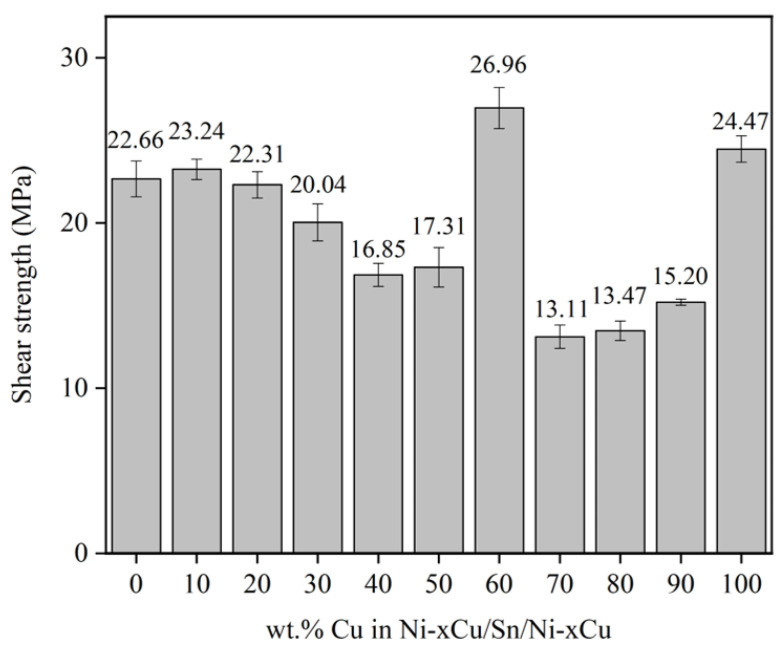
The shear strength of the Ni-xCu/Sn/Ni-xCu joints aged for 48 h.

Figure 6 shows the shear strength of the Ni-xCu/Sn/Ni-xCu joints aged for 48 h. A significant correlation exists between the mechanical properties of joints and the Cu content, exhibiting an initial decrease followed by an increase, except for a significant enhancement at a Cu content of 60 wt.%. Specifically, the measured average shear strength of the Ni-60Cu/Sn/Ni-60Cu joint is 26.96 MPa, slightly surpassing the Cu/Sn/Cu joint, which is 24.47 MPa.

**Figure 7:**
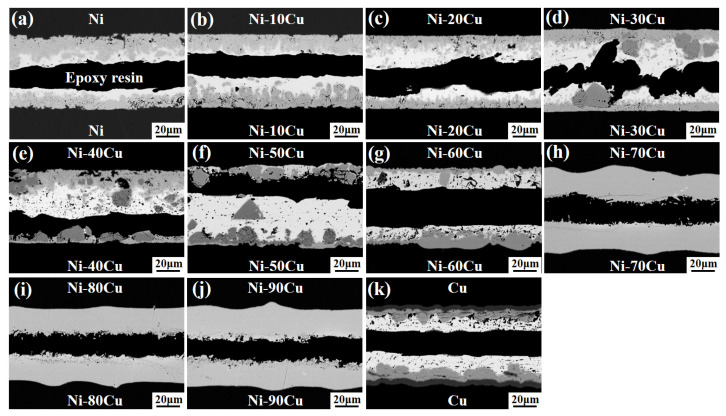
The fracture images of the Ni-xCu/Sn/Ni-xCu joints aged for 48 h: (**a**) 0, (**b**) 10, (**c**) 20, (**d**) 30, (**e**) 40, (**f**) 50, (**g**) 60, (**h**) 70, (**i**) 80, (**j**) 90, (**k**) 100.

Figure 7 shows the fracture images of the Ni-xCu/Sn/Ni-xCu joints aged for 48 h. The position and morphology of the fracture are closely associated with the phase structure and distribution of the IMCs in the joints. The fracture occurs inside the Sn solder with a smooth surface when the Cu content ≤20 wt.%. The fracture in the case of 30 wt.% Cu content primarily takes place within the Sn solder, partially along with the (Cu,Ni)_6_Sn_5_ particle phase, leading to a jagged surface. The fracture occurs along the interface between the (Cu,Ni)_6_Sn_5_ particle phase and the Sn solder as the Cu content reaches 40 and 50 wt.%. When the Cu content is increased to 40 and 50 wt.%, the fracture occurs at the interface between the (Cu,Ni)_6_Sn_5_ particle phase and the Sn solder. When the Cu content reaches 60 wt.% or pure Cu substrate is used, the fracture position reverts back to inside the Sn solder, displaying a smooth surface. In the case of Cu contents ranging from 70 to 90 wt.%, the Sn solder is depleted, and the fracture occurs at the center of the full-IMC joint, resulting in a rough surface.

**Figure 8:**
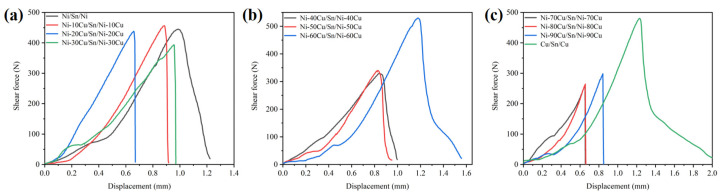
The displacement-shear force curves of the Ni-xCu/Sn/Ni-xCu joints aged for 48 h: (**a**) 0 ≤ x ≤ 30, (**b**) 40 ≤ x ≤ 60, (**c**) 70 ≤ x ≤ 100.

The failure mode of aged joints was further studied, and a comprehensive analysis of mechanical properties and failure mechanisms was conducted in conjunction with micrographs of fracture sections. Figure 8 shows the displacement-shear force curves of the Ni-xCu/Sn/Ni-xCu joints aged for 48 h. In Figure 8a, the joint fracture curves exhibit a steep decline for pure Ni substrate, indicating a combination of brittle and ductile fractures. However, in the case where the Cu content incorporated into Ni ranges from 10 to 30 wt.%, the fracture curve of the joints decreases vertically, indicating a transition to brittle fracture mode. In Figure 8b, with the Cu content ranging from 40 to 60 wt.%, the failure mode of the joints shifts to a hybrid fracture. The transition in failure mode is correlated with the microstructure, phase composition, fracture position, and grain size of the Sn solder, which can be evidenced in Figure 5 and Figure 7. In Figure 8c, the failure mode of the joints reverts back to brittle fracture when the Cu content increases to 70–90 wt.%, while the failure mode of the joint remains a hybrid fracture for the pure Cu substrate.

The evolution mechanism of the shear strength of aging joints can be comprehensively analyzed according to the results shown in Figure 7 and Figure 8. The shear strength decrease of the joints with Cu content from 10 to 30 wt.% is mainly caused by the failure mode transition of joint to brittle fracture. This transformation mechanism originates from the exfoliation of a large number of (Ni,Cu)_3_Sn_4_ particles into the Sn solder, which leads to increased solder brittleness [26]. When the Cu content in the joint is 40 and 50 wt.%, the shear strength is further reduced. This is because the existence of the (Cu,Ni)_6_Sn_5_ particle phase can promote the fracture that occurs preferentially at its interface with the solder, thereby reducing the mechanical properties of the joint. When the Cu content is 60 wt.%, the joint shear strength increases significantly. This is not only because the IMCs at the Ni-60Cu/Sn interface are single-phase with fine grains, but also because the IMCs have a slower growth rate compared to Cu/Sn. When the Cu content is 70 to 90 wt.%, the joint quickly forms a full IMC, and there is a large number of shrinkage cavities in the center of the weld, resulting in the transition of the joint to brittle fracture with the lowest shear strength.

## 4. Conclusions

The microstructure evolution and mechanical properties of the Ni-xCu/Sn interface during the solid-state reaction were studied at 200 °C with different Cu content and aging time. The main results are as follows:

1. The phase composition of IMCs exhibited a Cu content-dependent transformation from the (Ni,Cu)_3_Sn_4_ phase to the (Cu,Ni)_6_Sn_5_ phase at the Ni-xCu/Sn interface, and a Cu_3_Sn layer was observed exclusively at the Cu/Sn interface;
2. The morphology of the IMC layer underwent a transition from a continuous layer to a layer with particles above it, and finally to a continuous layer again. The IMC layer thickness at the Ni-xCu/Sn interface remains relatively consistent, except for cases where a full-IMC layer was formed at Cu contents of 80 and 90 wt.%;
3. The shear strength of the Ni-xCu/Sn/Ni-xCu soldering joints after aging exhibited an initial decrease followed by an increase, except for a significant enhancement at a Cu content of 60 wt.%. The decline in mechanical properties of the aging joints could be attributed to solder brittleness, the growth of (Cu,Ni)_6_Sn_5_ particles, and the presence of shrinkage cavities in the center of full-IMC welds. Notably, at a Cu content of 60 wt.%, the (Cu,Ni)_6_Sn_5_ phase exhibited a slow growth rate with refined grains at the interface, resulting in improved mechanical properties of the joint.

## Figures and Tables

**Table 1:** The EDS detection results on the designated positions marked in Figure 4.

Specimens	Positions	Element Component (at.%)			Phase
		Sn	Ni	Cu	
Ni/Sn	1	57.40	42.60	/	Ni_3_Sn_4_
Ni-10Cu/Sn	2	58.23	38.10	3.67	(Ni,Cu)_3_Sn_4_
Ni-20Cu/Sn	3	57.15	35.02	7.83	(Ni,Cu)_3_Sn_4_
Ni-30Cu/Sn	4	57.38	36.05	6.56	(Ni,Cu)_3_Sn_4_
	5	46.45	21.44	32.11	(Cu,Ni)_6_Sn_5_
Ni-40Cu/Sn	6	57.23	34.56	8.21	(Ni,Cu)_3_Sn_4_
	7	45.65	23.28	31.07	(Cu,Ni)_6_Sn_5_
Ni-50Cu/Sn	8	57.57	33.65	8.79	(Ni,Cu)_3_Sn_4_
	9	46.56	20.42	33.02	(Cu,Ni)_6_Sn_5_
Ni-60Cu/Sn	10	46.39	16.67	36.94	(Cu,Ni)_6_Sn_5_
	11	45.97	18.68	35.35	(Cu,Ni)_6_Sn_5_
Ni-70Cu/Sn	12	46.47	15.18	38.35	(Cu,Ni)_6_Sn_5_
Ni-80Cu/Sn	13	45.84	8.18	45.99	(Cu,Ni)_6_Sn_5_
Ni-90Cu/Sn	14	46.92	6.67	46.41	(Cu,Ni)_6_Sn_5_
Cu/Sn	15	46.48	/	53.52	Cu_6_Sn_5_
	16	73.71	/	26.29	Cu_3_Sn

## Data Availability

The data presented in this study are available on request from the corresponding author.
